# Genetic Polymorphisms in Glutathione (GSH-) Related Genes Affect the Plasmatic Hg/Whole Blood Hg Partitioning and the Distribution between Inorganic and Methylmercury Levels in Plasma Collected from a Fish-Eating Population

**DOI:** 10.1155/2014/940952

**Published:** 2014-02-18

**Authors:** Andréia Ávila Soares de Oliveira, Marilesia Ferreira de Souza, André van Helvoort Lengert, Marcelo Tempesta de Oliveira, Rossana Batista de Oliveira Godoy Camargo, Gilberto Úbida Leite Braga, Ilce Mara de Syllos Cólus, Fernando Barbosa, Gustavo Rafael Mazzaron Barcelos

**Affiliations:** ^1^Department of Clinical Analyses, Toxicology and Food Sciences, School of Pharmaceutical Sciences of Ribeirão Preto, University of São Paulo, Avenida do Café s/n°, 14040-903 Ribeirão Preto, SP, Brazil; ^2^Department of General Biology, Center for Biological Sciences, State University of Londrina, Rodovia Celso Garcia Cid km 380, 86051-990 Londrina, PR, Brazil

## Abstract

This study aims to evaluate the effects of polymorphisms in glutathione (GSH-) related genes (*GSTM1*, *GSTT1*, *GSTP1*, *GCLM*, and *GCLC*) in the distribution of Hg in the blood compartments in humans exposed to methylmercury (MeHg). Subjects (*n* = 88), exposed to MeHg from fish consumption, were enrolled in the study. Hg species in the plasma compartment were determined by LC-ICP-MS, whereas genotyping was performed by PCR assays. Mean total Hg levels in plasma (THgP) and whole blood (THgB) were 10 ± 4.2 and 37 ± 21, whereas mean evels of plasmatic MeHg (MeHgP), inorganic Hg (IHgP), and HgP/HgB were 4.3 ± 2.9, 5.8 ± 2.3 µg/L, and 0.33 ± 0.15, respectively. *GSTM1* and *GCLC* polymorphisms influence THgP and MeHgP (multivariate analyses, *P* < 0.050). Null homozygotes for *GSTM1* showed higher THgP and MeHgP levels compared to subjects with *GSTM1* (THgP *β* = 0.22, *P* = 0.035; MeHgP *β* = 0.30, *P* = 0.050) and persons carrying at least one T allele for *GCLC* had significant higher MeHgP (*β* = 0.59, *P* = 0.046). Also, polymorphic *GCLM* subjects had lower THgP/THgB than those with the nonvariant genotype. Taken together, data of this study suggest that GSH-related polymorphisms may change the metabolism of MeHg by modifying the distribution of mercury species iin plasma compartment and the HgP/HgB partitioning.

## 1. Introduction

Mercury (Hg) exposure during early life is associated with impaired neurodevelopment [1–4] and later in life, with adverse effects on the cardiovascular system [5, 6]. Fish is the major source of Hg exposure in fish-eating communities, where the methylmercury (MeHg) is the main species and presents the highest toxicity [7]. In the Amazonian region, several riverside populations, who have fish as the main source of proteins, are chronically exposed to high levels of MeHg [8].

For biomonitoring Hg exposure, several biomarkers have been proposed [9–12]. For instance, urinary levels of Hg frequently estimate the level of exposure to Hg vapors or inorganic Hg (IHg) whereas blood Hg and/or hair Hg predicts MeHg exposure [13].

According to Lorscheider et al. [14], most of the MeHg in our body is attached to hemoglobin (Hb) in red blood cells, with a small fraction coupled to GSH that contributes approximately to 1% of all circulating blood MeHg. Therefore, the use of whole blood as a biomarker of Hg exposure barely reflects available Hg, since only 1% or less of the metal is in the mobile form (Hg-cysteine) that effectively reaches the target organs [15]. Due to a higher portion of unbound Hg in comparison to blood, plasmatic Hg may be considered a fraction more freely available for exchange with target tissues than Hg levels in whole blood [16]. In this context, some recent publications demonstrate interesting and relevant associations between plasmatic Hg and outcomes in MeHg exposed subjects [17, 18].

On the other hand, it is generally accepted that Hg predominates in plasma in its inorganic form [19, 20]. However, data supporting this judgment are very limited and based on populations exposed to very low levels of Hg and only exposed to IHg. Moreover, data on the total plasmatic Hg/whole blood Hg (THgP/THgB) partitioning are totally inexistent for MeHg exposed populations.

Hg elimination in humans is linked to the glutathione (GSH) detoxification system in bile and several enzymes of this pathway may be involved in its elimination, such as the glutamyl-cysteine-ligases (GCLs) and the glutathione-S-transferases (GSTs) [21]. Many GSH-related enzymes are highly polymorphic and epidemiological studies have found that some polymorphisms in GSH-related genes are associated with the metabolism of Hg [22–28] and result in differences of Hg retention. Moreover, since the polymorphisms of GSH-related genes are associated to differences in Hg retention in the body, these genetic variations may also modify the partitioning of Hg between red cells and plasma for a given Hg whole blood which can be also associated with different toxicologically labile fraction of circulatory Hg.

Then, the aim of the present study was to evaluate the effects of polymorphisms in (*GSTM1*, *GSTT1*,* GSTP1*, *GCLM, *and *GCLC*) on the distribution of mercury species (MeHg and IHg) in the plasma compartment, as well as on the differences in the THgP/THgB partitioning in a group of persons exposed to the metal via consumption of contaminated fish in the Amazonian region of Brazil.

## 2. Materials and Methods

### 2.1. Study Design and Population

We carried out a cross-sectional study with participants from several riverside communities situated on the banks of the Tapajós River, one of the major tributaries of the Amazon River. Recruitment was conducted in 12 villages through a door-to-door invitation followed by community meetings. 88 subjects agreed to participate in the study.

The riverside communities of Brazilian Amazon are different from other populations of Brazil. The persons have a very specific diet; around 80% of the protein intake comes from fish and the consumption of vegetables and fruits is basically restricted to the region's typical ones [29]. In most of the villages of the study, there are no industrial activities or roads or vehicles, although a few motorized boats are used for fishing and transportation. Moreover, there is no gold-mining close to these communities and no participants reported to have amalgam fillings. Therefore, the only source of Hg exposure is through the intake of contaminated fish, where Hg is predominantly found in MeHg form [8].

Villagers' data were collected using two interviewer-administered questionnaires. One questionnaire covered sociodemographic, life-style, and health information (gender, age, village of residence, place of birth, length of time in the region, educational level, subsistence activities, exposure to other contaminants, frequency and quantity of smoking, drinking and drug habits, medical history and medication). The second was a 7-day recall food consumption frequency questionnaire. For fish consumption, a list was prepared which included most of the fish species present in the region. For each day, participants indicated the number of meals containing fish as well as the fish species that were consumed. Anthropometric measurements (weight, height, and waist circumference) were also taken by a trained technician.

Written consent was provided by all study participants. This study was approved by Ethics Committee of the University of São Paulo at Ribeirão Preto (Brazil), protocol number CEP/FCFRP-71.

### 2.2. Samples Collection and Hg Analyses

Blood samples were collected in trace metal-free vacuum tubes (BD Vacutainer, Franklin Lakes, NJ, USA) containing heparin. Plasma Hg species were determined using HPLC-ICP-MS (ELAN DRCII, SCIEX, Norwalk, CT, USA), according the method proposed by Souza et al. [30]. Samples (250 *μ*L; in triplicate) were placed in 15 mL polypropylene test tubes with 2.75 mL of a solution containing 0.10% v/v HCl + 0.050% m/v L-cysteine + 0.10% v/v 2-mercaptoethanol and then sonicated for 15 min in ultrasonic bath (UNIQUE, Brazil). The resulting solution was centrifuged and filtered through 0.20 *μ*m Nylon filters (Millipore, USA) and 100 *μ*L were injected in HPLC-ICP-MS. All separations were performed at room temperature under isocratic conditions. The isocratic mobile phase was 3% v/v methanol + 97% v/v (0.5% v/v 2-mercaptoethanol + 0.05% v/v formic acid). The flow rate was 1.2 mL/min. Data evaluation was performed using Chromera software (version 2.1.0.1631) supplied with the instrument, and quantifications were based on peak areas by external calibration. This method determines three species of Hg, but only MeHg and IHg were detected in the plasma samples that we analyzed.

Hg determination of quality control was guaranteed by analyzing standard reference materials from the U.S. National Institute of Standards and Technologies (NIST 966-Toxic Metals in Bovine Blood, certified value 31 ± 1.7 *μ*g/L and mean found value 31 ± 0.30 *μ*g/L). Moreover, various secondary reference materials, provided by the National Institute of Public Health of Quebec, Canada (INSP External Quality Assessment Scheme (EQAS) for Trace Elements in Blood, Plasma and Hair), were also analyzed. For these reference materials recoveries of Hg were between 93 and 105% (based on target values).

### 2.3. DNA Isolation and Genotyping

Genomic DNA was extracted from peripheral blood using the Easy-DNA kit (Invitrogen, Carlsbad, CA, USA) according to the manufacturer's instructions and stored at −20°C until analyses. *GSTM1* and *GSTT1* deletions were genotyped using multiplex-PCR as described by Abdel-Rahman et al. [31], with *CYP1A1* (exon 7) as an internal control to ensure good quality of the DNA. The primers, dNTPs, *Taq* polymerase, and magnesium chloride, were obtained from Invitrogen (Carlsbad, CA, USA). After amplification, PCR products were subjected to electrophoresis on a 2.0% agarose gel (Invitrogen, Carlsbad, CA, USA) and visualized using ethidium bromide (Sigma-Aldrich, St. Louis, MO, USA). DNA from samples positive for the *GSTM1* and *GSTT1* genes yielded bands of 215 and 480 bp, respectively, while the internal positive control (*CYP1A1*) PCR product yielded a 312 bp fragment. *GSTP1 *Ile^105^Val (rs1695), *GCLM*-588 (rs41303970), and *GCLC*-129 (rs17883901) were genotyped by real-time PCR using TaqMan assays (Applied Biosystems, Carlsbad, CA, USA) as described by Custodio et al. [24] on a Quantica Real Time PCR System (TECHNE; Staffordshire, UK).

### 2.4. Statistical Analysis

Hardy-Weinberg equilibrium was analyzed with the conventional Chi-square test. Age, fish intake, and biomarkers of Hg were analyzed as continuous variables; gender, genotypes, alcohol consumption, and smoking as categorical variables. We considered participants that consumed at least five drinks per week as alcohol users and smokers those who smoke at least five cigarettes per day for the last five years.

Correlations (Spearman's; rho) were performed in order to examine the associations between age, gender, fish intake, alcohol consumption, smoking, and Hg biomarkers. After that, Student's *t*-tests were performed to assess the variations between the Hg biomarkers among the different genotypes.

Finally, multivariate general linear models were employed to evaluate the influence of genetic effects on Hg biomarkers. In order to adjust for other variables influencing Hg concentrations, the impacts of age, gender, body mass index, fish intake, alcohol consumption, and smoking on Hg levels were analyzed in a univariate model and variables were included in the multivariate model if they had a *P* value <0.20, that is, in the present study, age, gender, and fish intake. All Hg biomarkers were ln-transformed through the analyses, because the nontransformed values were not normally distributed.

Results were defined as statistically significant for a value of *P* ≤ 0.050. Analyses were performed using SPSS 20 Statistics software (IBM; Armonk, NY, USA).

## 3. Results

### 3.1. General Characteristics

Sociodemographic characteristics and Hg concentrations for all participants enrolled are described in Table 1. The age ranged between 15 and 80 years; the distribution between the sexes was homogenous and fish consumption (portion of fish per day; 150–200 g per meal) varied from one to four portions per day (2.5 ± 1.5). Alcohol was consumed by 34% and 23% of the study participants were smokers. No participants reported to have amalgam fillings. THgB, THgP, MeHgP, and IHgP were 37 ± 21, 10 ± 4.3, 4.3 ± 2.9, and 5.8 ± 2.3 *μ*g/L, respectively; THgP/THgB ranged from 0.13 to 0.91.

Table 2 presents genetic background data and comparative allele frequencies of Caucasians and Africans found in earlier studies (http://www.hapmap.org/, populations CEU; CEPH (Utah residents with ancestry from northern and Western Europe; and YRI; Yoruba in Ibadan, Nigeria)), because the study population has a genetic background from European colonizers and African slaves. All allelic frequencies were in Hardy-Weinberg equilibrium; in general, for the five polymorphisms analyzed, the genetic/allelic frequencies were more similar to those found in the reference African population than the European ones.

### 3.2. Correlations between Fish Intake, Lifestyle, and Hg Biomarkers

**Figure 1:**
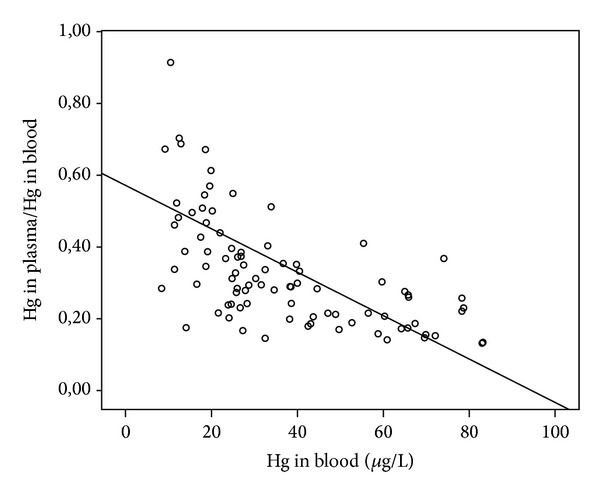
Correlation between total mercury in blood and the Hg in plasma/Hg in blood (Spearman's; *r*_*s*_ 0.71, *P* < 0.0010).

Correlations between the variables enrolled in the present work are present in Table 3. Interestingly, here, fish consumption was only positively correlated to MeHgP (*r*_*S*_ = 0.32, *P* < 0.0010). Moreover, THgB, THgP, MeHgP, and IHgP were highly correlated and the highest correlation was found between THgP and MeHgP (*r*_*S*_ = 0.81, *P* < 0.0010). Age was positively correlated to THgB, THgP and IHgP, while no correlations were found between Hg biomarkers and gender. Also, a negative correlation was found between THgB and THgP/THgB (*r*_*S*_ = 0.71, *P* < 0.0010) (Figure 1). No correlations were found between smoking and alcohol consumption and Hg biomarkers.

### 3.3. Genetic Effects and Hg Partitioning between Plasma and Whole Blood

Table 4 shows the concentrations of Hg biomarkers among the different genotypes. It can be seen that participants with the *GSTM1* null genotype had higher levels of THgB and IHgP than subjects that expressed the enzyme. Also, persons carrying at least one allele T for *GCLC* had higher levels of THgP, MeHgP and IHgP compared to those with the nonvariant genotype, while polymorphic *GLCM* individuals had lower percentage of THgP in the blood stream.

Table 5 summarizes the genetic effects obtained from multivariate regressions for concentrations of Hg biomarkers, adjusted for fish intake, gender, and age. *GSTM1 *and *GCLC* polymorphisms modified THgP and MeHgP (multivariate analyses, *P* < 0.050). Null homozygotes for *GSTM1* accumulated more THgP and MeHgP compared to subjects with *GSTM1* (*β* = 0.22, *P* = 0.035 and *β* = 0.30, *P* = 0.050, resp.).

**Figure 2:**
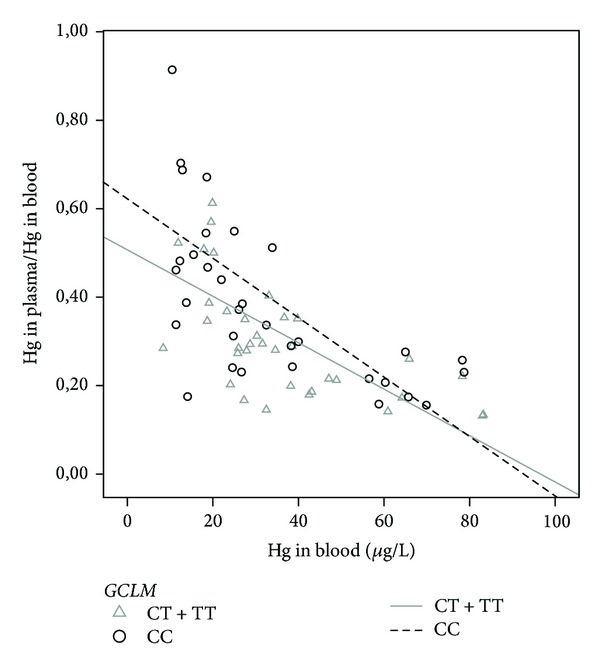
Hg in blood as a function of Hg in plasma/Hg in blood for *GCLM* genotypes. The regression lines do not reflect adjustments for age and gender as described in the text.

Genetic effects were also seen concerning *GCLs *polymorphisms. Persons who are carrying at least one T allele for *GCLC *had higher THgP as well as MeHgP concentration (*β* = 0.45, *P* = 0.046; *β* = 0.69, *P* = 0.038, resp.). Interestingly, *GCLM *polymorphism altered the relation between the levels of THgP and THgB; that is, participants who carried the polymorphic allele tended to have lower levels of THgP than those with the nonvariant genotype (*β* = −0.21, *P* = 0.050) (Figure 2).

## 4. Discussion

The present work shows that polymorphisms in glutathione-related genes modify the relationship between exposure to the metal and Hg species in plasma in a population highly exposed, via fish intake.

Correlations between fish intake and MeHgP were found, indicating that the populations are exposed predominantly to MeHg. However, significant correlations were not found between fish consumption and THgB, THgP, and IHgP. Accessing the association between the exposure to MeHg and retention requires estimates of both. The exposure to MeHg depends on both the intake of fish and the concentration of the metal in the fish. Here, only data about fish intake collected by questionnaire was used and therefore, the variation of Hg levels in the fish, which was not estimated in this study, can be seen in the differences found in the correlations between fish consumption and Hg biomarkers (Table 3). Our group has previously found that MeHgP are associated with fish intake, while IHgP is not; the increase of IHgP was attributed to age and it is supposed that other processes of demethylation may be also evolved (data not published). However, we believe that other variations, such as genetic effects, are also associated with Hg metabolism and may explain part of this scenario.

According to the World Health Organization [33], after the ingestion of fish contaminated, approximately 95% of Hg is absorbed in the gastrointestinal tract resulting in 5% of Hg in the blood compartment and the ratio between THgP and THgB is 1/20 [34]. However low, Hg in plasma is the bioavailable fraction and may be related to increase of adverse health effects. However, nothing has been explored considering the effects of chemical forms of Hg, mainly in the plasma compartment as well as their usefulness to assess toxicological risks.

We observed a negative correlation between THgB and THgP/THgB; that is, with increase of THgB, a decrease in the ratio THgP/THgB is observed, indicating that even with the increase of THgB, saturation effects of erythrocytes do not occur, and therefore, there is no Hg mobilization of erythrocytes to the plasma fraction. In blood, MeHg is predominantly bound to erythrocytes, while IHg is mostly in plasma. When the exposure is to IHg, the increase of THgP is independent of the concentration of THgB, since IHg binds weakly to red blood cells [15, 20, 22]; our data give further support to these previous findings, since we observed a strong correlation between THgB and THgP. Therefore, our results suggest that determination of Hg in total blood may not predict directly the toxicity in individuals predominantly exposed to MeHg and alternative approaches to assess the toxicological effects associated with MeHg exposure should be strongly encouraged.

As mentioned above, THgP levels are highly correlated to MeHgP; therefore, the changes in THgP probably are associated to the alterations in MeHgP. We found higher concentrations of MeHgP among *GSTM1* null participants, which may be related to lower MeHg-conjugating activity, lower MeHg excretion, and a higher MeHg retention.

Earlier findings of our group have also found associations between THgB and *GSTM1* polymorphisms [22, 23], which were not supported in the present study; these differences between our studies may be explained by the size of the sample, which is reduced here, but not limiting for gene-environment interactions studies. In studies of Swedish populations exposed to MeHg via fish intake and to IHg by gold mining activity, Custodio et al. [24, 25] found no associations between *GSTM1* and concentrations of Hg in blood. On the other hand, Lee et al. [27] evaluated 417 pregnant women of North Korea exposed to MeHg via fish consumption and observed that women who had homozygous deletion for *GSTM1* had higher Hg levels in blood, either during the early or late pregnancy (Hg in blood 3.7 and 3.3 *μ*g/L, resp.), than persons with the gene.

The different results between the studies might be related to different levels of MeHg exposure. The studies of Gundacker et al. [35], Custodio et al. [24], and Engström et al. [28] were performed on study participants with lower MeHg exposure than the study carried by Lee et al. [27] and our earlier study [22, 23]. Another hypothesis is that the differences also may be explained, in some extent, to the biomarker used in the studies; however, as mentioned above, currently there is no data concerning assessment of genetic effects on Hg species in plasma.

We did not find association between *GSTT1* polymorphism and THgP, MeHgP, and IHgP levels. An earlier work showed the *GSTT1* null polymorphisms are associated with accumulation of Hg in individuals exposed to EtHg via vaccines [36]. According to Custodio et al. [24], the dealkylation of EtHg occurs faster than MeHg and therefore, the genetic effects may thus affect the elimination of IHg, a point which was not observed in a further study of the same group [25] and, also, in the present work. However, in a previous work, we observed an association between THgB and *GSTT1* polymorphisms [22] in the same studied population. Thus, these data bring some pieces of evidence that the genetic effects of *GSTT1* may be more associated with the bioavailable MeHg or other organic forms than the levels of exposure to Hg as well as the exposure to IHg.

Also, in the present work, association between polymorphism of *GSTP1* and Hg biomarkers was not found. Actually, a recent experimental study demonstrated that the Ile allele is more sensitive to Hg exposure. Goodrich and Basu [37] evaluated the activity of allozymes of *GSTP1 *towards IHg and MeHg and found that the *GSTP1* Val allozyme was less sensitive to inhibition induced by treatment of high doses of Hg than the Ile allozyme. In support of this data, previous *in vitro* studies have suggested that *105Val amino acid may confer protection against Hg-induced inhibition due to structural changes in cysteine residues which may impact the ability of Hg to bind and, consequently, inhibit the enzyme [38, 39].

*GCLM *polymorphism did not impact the concentrations of Hg biomarkers but altered the percentage of THgP in the blood stream (as seen in Figure 2). A previous work of our group [23] showed that polymorphic individuals for *GCLM* had lower THgB levels than those who carried at least one C-allele. However, although these polymorphic individuals had lower THgB levels, they tended to have more THgP, which may modulate the adverse health effects related to MeHg exposure. Moreover, variation in *GCLC *gene influences the levels of MeHgP. Here, we found that T-carriers for *GCLC *allele had significant higher levels of MeHgP than subjects with the nonvariant genotype. These data suggest that polymorphisms in *GCLs *may be related to biomarkers for Hg as well the species of the metal; also, interindividuals variations may be considered. For example, Engström et al. [28] found that Swedish persons with GCLM CC genotype had lower THgB concentrations than those who carried at least one T allele, whereas Custodio et al. [24] did not find any association at lower MeHg exposure, in same studied populations. Interestingly, in the same work, authors showed that carriers of T allele for *GCLC* had lower concentrations of Hg in erythrocytes than CC subjects, a contradictory result compared to our present.

To our knowledge, this work is the first to investigate the genetic effects of Hg species on plasma. Taken together, our results indicate that GSH-related polymorphisms may influence levels of Hg fractions in plasma, which may modulate Hg-induced toxicity. However, further studies concerning both Hg species in plasma as well as genetic effects are necessary for a better elucidation of the mechanisms evolved in Hg species metabolism.

## Figures and Tables

**Table 1:** General characteristics of riverside persons living in Amazonian region, Brazil.

	*N*	Mean ± SD	Median	Range
Participants	88	—	—	—
Age (years)	88	41 ± 16	41	15–80
Female/male	37/51	—	—	—
Body mass index	88	25 ± 4.6	24	17–42
Portion fish/day^a^	86	2.5 ± 1.5	3.0	1.0–4.0
1	15	—	—	—
2	26	—	—	—
3	13	—	—	—
4	32	—	—	—
Smoking (yes)	88 (20)	—	—	—
Alcohol (yes)	88 (30)	—	—	—
THgB^b^ (µg/L)	88	37 ± 21	29	8.4–83
THgP^c^ (µg/L)	88	10 ± 4.3	9.6	2.4–27
THgP/THgB^f^	88	0.33 ± 0.15	0.29	0.13–0.91
MeHgP^d^ (µg/L)	88	4.3 ± 2.9	3.6	0.67–18
IHgP^e^ (µg/L)	88	5.8 ± 2.3	5.7	1.1–13

^a^One portion of fish: 150–200 g; two participants did not answer the questionnaire concerning fish intake.

^b^Total Hg in blood.

^c^Total Hg in plasma.

^d^Methylmercury in plasma.

^e^Inorganic Hg in plasma.

^f^Ratio between total Hg in plasma and total Hg in blood.

**Table 2:** Genotype, allele frequencies of *GSTM1*, *GSTT1*, *GSTP1*, *GCLM*, and *GCLC* polymorphisms of riverside persons living in the Amazonian region of the Tapajós River, Brazil.

Genotypes	Genotype frequencies		MAF^a^			Reference MAF	
						European	African
*GSTM1 *	Present	Null	—	—	Null^c^	Null^c^
Deletion	0.66	0.34	—	—	0.13–0.54	0.47
*GSTT1 *	Present	Null	—	—	Null^c^	Null^c^
Deletion	0.60	0.40	—	—	0.11–0.28	0.37
*GSTP1* (Ile^105^Val)	Ile/Ile	Ile/Val + Val/Val	Val (G)	HWE^b^	Val (G)^d^	Val (G)^g^
rs1695	0.37	0.63	0.40	Yes	0.42	0.39
*GCLM-*588 (C/T)	CC	CT + TT	T	HWE	T^e^	T^h^
rs41303970	0.48	0.52	0.33	Yes	0.10	0.25
*GCLC*-128 (C/T)	CC	CT + TT	T	HWE	T^f^	T^f^
rs17883901	0.95	0.050	0.030	Yes	0.060	0.010

^a^Minor allele frequency.

^b^HWE: Hardy-Weinberg equilibrium. For the *GSTM1* and *GSTT1* deletions it was not possible to calculate HWE, because the methodology used does not distinguish between hetero- and homozygous genotypes.

^c^Reference values from Mo et al. [32].

^d^Reference values for ss1390210 from HapMap-CEU.

^e^Reference values for ss230641266 from pilot_1_CEU_low_coverage_panel.

^f^Reference values for ss66860389 from CEU_GENO_PANEL; YRI_GENO_PANEL.

^g^Reference values for ss1390210 from HapMap-YRI.

^h^Reference values for ss218528824 from pilot_1_YRI_low_coverage_panel.

**Table 3:** Correlations (Spearman's; *r*_*s*_) between age, gender, body mass index (BMI), alcohol consumption, smoking, total Hg in blood (THgB) and total Hg, methylmercury, and inorganic Hg in plasma (THgP, MeHgP, and IHgP, resp.).

	Age	Gender	BMI	Fish intake	Alcohol	Smoking	THgB	THgP	THgP/THgB	MeHgP	IHgP
Age	—	−0.13	0.18	−0.17	−0.20	−0.16	0.27*	0.21*	−0.16	0.059	0.27*
Gender^a^	−0.13	—	0.051	−0.32**	−0.030	0.19	−0.11	−0.036	0.13	0.057	−0.12
BMI	0.18	0.051	—	−0.15	0.12	0.022	−0.072	0.0090	0.071	−0.075	0.12
Fish intake^b^	−0.17	−0.32	−0.15	—	0.12	−0.030	0.20	0.21	−0.075	0.32**	−0.0090
Alcohol	−0.20	−0.030	0.12	0.12	—	0.16	−0.077	0.086	0.17	0.096	0.041
Smoking	−0.16	0.19	0.022	−0.030	0.16	—	−0.043	−0.12	−0.032	−0.098	−0.060
THgB	0.27*	−0.11	−0.072	0.20	−0.077	−0.043	—	0.66**	0.71**	0.61**	0.48**
THgP	0.21*	−0.036	0.0090	0.21	0.086	−0.12	0.66**	—	0.040	0.81**	0.77**
THgP/THgB	−0.16	0.13	0.071	−0.075	0.17	−0.032	0.71**	0.040	—	−0.11	0.11
MeHgP	0.059	0.057	−0.075	0.32**	0.096	−0.098	0.61**	0.81**	−0.11	—	0.32**
IHgP	0.27*	−0.12	0.12	−0.0090	0.041	−0.060	0.48**	0.77**	0.11	0.32**	—

^a^Female was considered as reference. ^b^Fisk intake ranged from one to four portions per day.

*Statistically significant *P* < 0.050; **statistically significant *P* < 0.010.

**Table 4:** Total Hg in blood (THgB) and total Hg, methylmercury, and inorganic Hg in plasma (THgP, MeHgP, and IHgP, resp.) as well as the ratio THgP/THgB among the different genotypes.

Genotypes	THgB	THgP	MeHgP	IHgP	THgP/THgB
	Mean ± SD^d^	Mean ± SD	Mean ± SD	Mean ± SD	Mean ± SD
*GSTM1 *	37 ± 21	10 ± 4.4	4.4 ± 2.5	5.9 ± 2.4	0.33 ± 0.16
Present	34 ± 18	9.6 ± 4.2	4.1 ± 2.8	5.5 ± 2.2	0.33 ± 0.14
Null	44 ± 24	12 ± 4.7*	5.1 ± 3.2	6.6 ± 2.5*	0.34 ± 0.18
*GSTT1 *	37 ± 21	10 ± 4.4	4.4 ± 2.9	5.9 ± 2.4	0.33 ± 0.16
Present	35 ± 22	9.9 ± 4.6	4.1 ± 3.1	5.7 ± 2.4	0.34 ± 0.17
Null	40 ± 19	11 ± 4.1	5.0 ± 2.7	6.0 ± 2.3	0.31 ± 0.13
*GSTP1* ^a^	38 ± 21	10 ± 4.0	4.3 ± 2.5	5.8 ± 2.3	0.32 ± 0.16
Ile/Ile	40 ± 22	11 ± 3.7	4.5 ± 2.3	6.1 ± 2.4	0.33 ± 0.15
Ile/Val + Val/Val	36 ± 20	9.8 ± 3.9	4.2 ± 2.6	5.6 ± 2.2	0.31 ± 0.16
*GCLM* ^b^	35 ± 20	9.7 ± 3.7	4.2 ± 2.3	5.6 ± 2.2	0.34 ± 0.16
CC	33 ± 21	10 ± 4.1	4.5 ± 2.4	5.7 ± 2.6	0.38 ± 0.16
CT + TT	36 ± 19	9.4 ± 3.2	3.9 ± 2.3	5.5 ± 1.7	0.30 ± 0.13*
*GCLC* ^c^	35 ± 19	9.9 ± 4.3	4.3 ± 3.0	5.6 ± 2.2	0.34 ± 0.15
CC	34 ± 20	9.7 ± 4.1	4.1 ± 2.9	5.5 ± 2.1	0.33 ± 0.16
CT + TT	42 ± 12	15 ± 6.2*	6.9 ± 3.2*	8.1 ± 3.1*	0.36 ± 0.13

^a^rs1695; ^b^rs41307970; ^c^rs17883901; ^d^arithmetic mean ± standard deviation (SD).

*indicates significant difference between the wild genotypes and the polymorphic ones (Student's *t*-test; *P* < 0.050).

**Table 5:** Multivariate regression parameters for the associations between genotype and total Hg in blood (THgB) and total Hg, methylmercury, and inorganic Hg in plasma (THgP, MeHgP, and IHgP, resp.) as well as the ratio THgP/THgB.

Genotypes	THgB^d^		THgP^d^		MeHgP^d^		IHgP^d^		THgP/THgB^d^	
	*β* ^*e*^	*P*	*β*	*P*	*β*	*P*	*β*	*P*	*β*	*P*
*GSTM1 *										
Present	—	—	—	—	—	—	—	—	—	—
Null	0.21	0.13	0.22	0.035	0.30	0.050	0.18	0.10	0.16	0.88
*GSTT1 *										
Present	—	—	—	—	—	—	—	—	—	—
Null	0.14	0.31	0.10	0.34	0.18	0.24	0.090	0.45	−0.040	0.71
*GSTP1* ^a^										
Ile/Ile	—	—	—	—	—	—	—	—	—	—
Ile/Val + Val/Val	−0.10	0.49	−0.12	0.27	−0.79	0.59	−0.11	0.36	−0.018	0.87
*GCLM* ^b^										
CC	—	—	—	—	—	—	—	—	—	—
CT + TT	0.12	0.38	−0.08	0.41	−0.16	0.29	−0.020	0.88	−0.21	0.050
*GCLC* ^c^										
CC	—	—	—	—	—	—	—	—	—	—
CT + TT	0.34	0.25	0.45	0.046	0.69	0.038	0.36	0.14	0.12	0.61

^a^rs1695; ^b^rs41307970; ^c^rs17883901; ^d^Natural ln-transformed.

^e^Unstandardized beta (*β*) coefficients for the *β*_1_ × genotype term (categorical) adjusted for covariates. The genotype denoted first is used as reference. Multivariate model: Hg biomarkers = *α* + *β*_1_ × genotype + *β*_2_ × fish intake + *β*_3_ × age + *β*_4_ × gender.
